# Rates and risk factors for human cutaneous anthrax in the country of Georgia: National surveillance data, 2008–2015

**DOI:** 10.1371/journal.pone.0192031

**Published:** 2018-02-07

**Authors:** Ana Kasradze, Diana Echeverria, Khatuna Zakhashvili, Christian Bautista, Nicholas Heyer, Paata Imnadze, Veriko Mitrskhulava

**Affiliations:** 1 National Centre for Disease Control and Public Health, Tbilisi, Georgia; 2 Branch of Battelle Memorial Institute in Georgia, Tbilisi, Georgia; 3 University of Washington, Seattle, Washington, United States of America; 4 US Walter Reed Army Institute of Research, Washington, D.C., United States of America; 5 David Tvildiani Medical University, Tbilisi, Georgia; Spectrum Health, UNITED STATES

## Abstract

**Introduction:**

Anthrax is endemic in the country of Georgia. The most common cutaneous anthrax form accounts for 95% of anthrax cases and often is self-resolving. Humans are infected from processing contaminated animal products, contacting sick animals, or by insect bites.

**Objective:**

We aimed to describe the burden of human cutaneous anthrax and associated risk factors using the national surveillance data.

**Methods:**

We extracted all human cutaneous anthrax cases from Electronic Integrated Disease Surveillance System (EIDSS) from 1 January 2008 to 31 December 2015. We conducted descriptive analyses to characterize the number of confirmed, probable and suspected cases by age groups, gender, ethnicity, year and geographic area.

**Results:**

Out of 911 reported cutaneous anthrax cases, 299 (33%) were rejected. Out of remaining 612 cases, 437 (71%), 172 (28%), and 3 (<0.004%) were classified as confirmed, probable and suspected cases of cutaneous Anthrax, respectively; 467 (76.3%) were male. Georgians accounted for 56% (343/612) of cutaneous anthrax cases. Handling animal products (aOR 4.36, 95% CI 2.61–7.26) and living near pastoralist routes (aOR 2.74, 95%CI 1.57–4.76) were associated with cutaneous anthrax.

**Conclusions:**

This study provides eight-year trends for cutaneous anthrax in humans in the country of Georgia. A comprehensive explanation for the observed rise and fall of the incidence rates of human cutaneous anthrax in 2008–2015 remains to be clarified but is likely associated with discontinuation of mandatory national livestock vaccination in 2008 coupled with weakened human and animal national health systems which were disrupted after the Soviet Union collapsed. Our analysis identifies living near pastoralist routes, handling animal products and travel to endemic areas within two weeks before the disease onset as risk factors for cutaneous anthrax. The evidence underscores the importance of One Health recommendations to activate anthrax awareness campaigns, supervise the destruction of known anthrax carcasses, record global position system coordinates of sites and disinfect infected soils and introduce a participatory health education tool on anthrax.

## Introduction

Anthrax is an acute infection caused by *Bacillus anthracis*, an aerobic gram-positive microorganism capable of producing spores, affecting both humans and animals. The sporulation makes *B*. *anthracis* long-lived and resistant to degradation in the environment [1]. In humans, anthrax presents in three forms: cutaneous, pulmonary and gastrointestinal. The most common cutaneous anthrax form accounts for 95% of anthrax cases and often is self-resolving. Humans are infected from processing contaminated animal products, contacting sick animals [2], or by insect bites [3]. Without treatment, the fatality rate of cutaneous anthrax is 20% [4].

Anthrax is endemic in the country Georgia. During World War II, 357 cutaneous anthrax human cases were reported across 247 identified Georgian foci [5]. From the late 1970s to early 1990s, the number of anthrax cases decreased in conjunction with a country-wide animal annual vaccination program; only 36 human cases were reported during 1985–1989 [5]. In 1989–1995 three cutaneous anthrax outbreaks occurred in humans associated with earthworks, including digging canals or graves in Gurjaani (22 cases in 1989), in Tskaltubo (38 cases in 1992) and Gardabani (30 cases in 1995) [5]. The outbreaks occurred about the time of the dissolution of the Soviet Union (1990–1998) which corresponds to the disruption in vaccine availability. In 1995, livestock vaccination was still mandatory and under the responsibility of the National Food Agency (NFA), which is responsible for animal health. Between 1995 to 2007, the number of cutaneous anthrax cases remained constant varying between 20–50 cases a year [5]. However, in 2007, livestock owners became financially responsible for vaccination of their livestock through private veterinarians [6]. After this policy change, an increase in human anthrax cases livestock through private veterinarians [6]. After this policy change, an increase in human anthrax cases. In late autumn of 2013, the NFA re-initiated vaccination of susceptible livestock at a limited number of high-risk locations in eastern Georgia. However, by 2015, the animal vaccination coverage was less than 20% in these areas.

There is limited data on the epidemiology and associated risk factors of human cutaneous anthrax in Georgia. We aim to describe the burden of human cutaneous anthrax in Georgia and associated risk factors using the national surveillance data. Our findings will serve as a baseline information to assess the renewed national animal vaccination program and to target future interventions.

## Methods

### Study design

We conducted a retrospective analysis of the national surveillance data of human cutaneous anthrax registered from 1 January 2008 to 31 December 2015 in the Electronic Integrated Disease Surveillance System (EIDSS) in the country of Georgia. Human and animal individual cases of notifiable diseases from all 68 municipal public health centers in the country [7]. Since 2006, EIDSS has been used as a surveillance tool for especially dangerous pathogens, including Anthrax and since 2012 became the single surveillance tool for all notifiable disease in the country. Furthermore, in collaboration with the NFA experts, a subset of towns, located next to animal corridors used by pastoralists was identified. The pastoralists constitute a mix of Georgian and Azeri population and a smaller number of Armenians [8].

### Definitions

All cases of cutaneous anthrax complied with standardized case definitions for cutaneous anthrax surveillance purposes.

**A suspected case of cutaneous anthrax** was defined as a patient with an acute illness with painless primary skin lesion surrounded by localized or extensive edema in one of the following stages: papule, vesicle, pustule (hemorrhagic), ulcer (flat, dry, with solid black eschar on the bottom, located on infiltrated basement and surrounded by hyperemic areola), painless solid black eschar [9].

**A probable case of cutaneous anthrax** was defined as a patient who met the suspected anthrax case definition and at least one of the following factors: within two weeks prior to onset of symptoms travel or live in a location where animal or human anthrax cases were reported; or handling animal products (meat, skin, leather or bones); or consumption of raw or undercooked meat; or cleaning of farms or territories where agricultural animals are/were kept; or bites of big blood-sucking insects (horseflies); or any work associated with soil in an area where ill animals were kept or buried or the immediate surrounding area; detection of gram-positive capsule and/or spore-developing bacillus in a microscopic study or smear, or a positive reaction to allergic skin test among persons with no anthrax vaccination or disease history, or a laboratory worker with potential occupational exposure to anthrax; or epidemiologic link with confirmed human or animal anthrax case [9].

**A confirmed case of cutaneous anthrax** was defined as a probable/suspected case with culture and identification of *B*. *anthracis* from clinical specimens or positive PCR results [9].

**A rejected case of cutaneous anthrax** was defined as a clinically compatible case without an epidemiological link and with negative laboratory results.

### Laboratory diagnosis

For initial diagnosis, vesicular fluid or eschar material samples were plated on sheep blood agar (SBA) media for culture isolation. DNA samples were extracted from cultures or broth enrichment media using Qiagen DNeasy Blood & Tissue Mini Kits per manufacturer’s instructions (QIAGen GMbH, Hilden, Germany) [10]. Final confirmation was performed by real-time PCR assay on Roche Light Cycler 2.0 using the BioFire diagnostic kit on *B*. *anthracis* Target 2 and *B*. *anthracis* Target 3 markers.

### Statistical analysis

For data analysis, we used SPSS version 21 (IBM Corporation, Chicago, US). We conducted descriptive analysis to characterize the number of confirmed, probable and suspected cases by age groups, gender, ethnicity, year and geographic area. To calculate annual cumulative incidence rates of cutaneous anthrax per 100 000 population by geographic location the national and ethnic population estimates were obtained for each of the eight years from all geographic locations from the National Statistics Office of Georgia (GeoStat). To identify how week-in-the-year may explain some of the observed variation in weekly counts of disease occurrence, we fitted an autoregressive integrated moving average (ARIMA) model [11]. The model permitted statistical testing of differences in year and week of the year (S1 File)

We used binary multiple logistic regression to identify statistically significant risk factors for cutaneous anthrax in humans in the country of Georgia. For the risk factor analysis, we used 2011–2015 surveillance data since only these years had complete and accurate risk factor data (>85%). Associations were expressed as adjusted odds ratios (aORs) adjusted for gender, age, ethnicity and geographic area. We analyzed towns near pastoralist routes versus other locations. We used the Hosmer-Lemenshow test to assess the adequacy of the model. The test was not significant (p-value = 0.129), and we considered the model well fitted. Our final model included age, gender, ethnicity, living near the pastoralist routes, handling animal products, travel to endemic areas within two weeks before the disease onset, and earthwork. To assess significance of the association 95% Confidence Interval (CI) was used. In this model, collinearity between predictor variables was assessed using a correlation matrix and chi-squared test between categorical variables. Ethnicity, a categorical variable with three categories was transformed into dummy variables (S2 File).

### Ethical statement

Any form of anthrax case is among notifiable disease list in the country of Georgia. The National Center for Disease Control and Public Health of Georgia considers data collection in EIDSS for human anthrax cases as the part of its routine surveillance. For the manuscript, we extracted the de-identified secondary data from EIDSS. Hence, the study was exempted from institutional review board approval.

## Results

During 2008–2015, 911 human cutaneous anthrax cases were registered in EIDSS, including 299 (33%) rejected cases. Of the remaining 612 cases, 437 (71%) were classified as confirmed, 172 (28%) as probable, and 3 (<0.004%) as suspected. (Table 1)

**Table 1:** Distribution of cutaneous anthrax cases in the country of Georgia by final case classification.

**Final case classification**	**Year**	**2008**	**2009**	**2010**	**2011**	**2012**	**2013**	**2014**	**2015**	**Sum**	**Total (%)**
	**Confirmed**	**37**	**25**	**18**	**62**	**111**	**103**	**38**	**43**	**437**	**71**
	**Probable**	**25**	**14**	**10**	**21**	**33**	**40**	**19**	**10**	**172**	**28**
	**Suspected**	**2**	**0**	**1**	**0**	**0**	**0**	**0**	**0**	**3**	**1**
	**Total**	**64**	**39**	**29**	**83**	**144**	**57**	**57**	**53**	**612**	

The highest number of cases was found in the age group of 30–59 (67.3%); 467 (76.3%) were male. Georgians accounted for 56% (343/612) of cutaneous anthrax cases, 36.4% were Azeris (223/612) and 5.2% were Armenians (32/612). We calculated annual cumulative incidence including confirmed, probable and suspected cases of cutaneous anthrax. In 2008, the annual incidence rate was 1.46/100 000. In 2010, it decreased to 0.65/100 000 and then increased to a peak of 3.20/100 000 in 2012. The incidence rate declined again to 1.27/100 000 in 2014 and 1.56/100 000 in 2015 (Table 2).

**Table 2:** Epidemiological characteristics of human cutaneous anthrax cases in the country of Georgia, 2008–2015.

Characteristics Total Cases (n = 612)	
Male, No. (%)	467 (76.3%)
Age, No. (%)	
0–14	14 (2.3%)
15–19	17 (2.8%)
20–29	81 (13.2%)
30–59	412 (67.3%)
60+	88 (14.4%)
**Ethnicity**	
Georgian	343 (56%)
Azeri	223 (36.4%)
Armenian	32 (5.2%)
Other	14 (2.4%)
**Distribution by year**	**Cumulative incidence rate/per 100,000**
2008	1.46
2009	0.89
2010	0.65
2011	1.86
**2012**	**3.20**
**2013**	**3.19**
2014	1.27
2015	1.56

**Fig 1:**
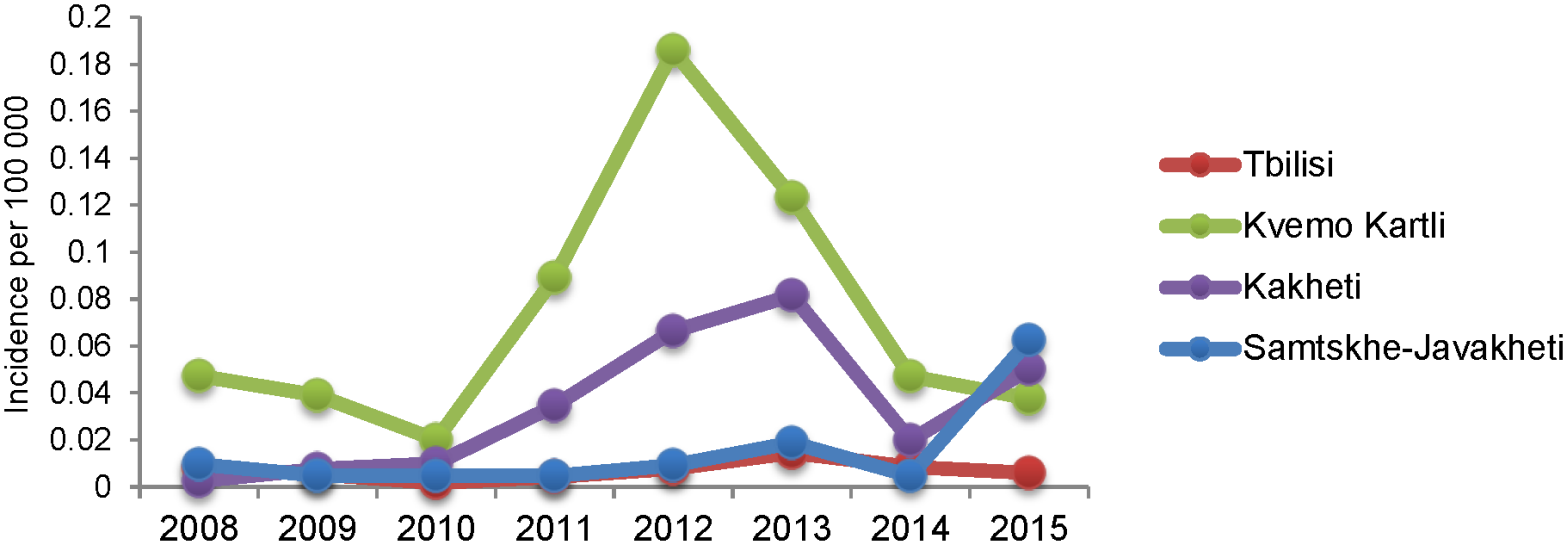
Incidence of human cutaneous anthrax in the most affected regions of Georgia, 2008–2015. National cumulative incidence rates from 2008 to 2015 was calculated per 100 000 population by geographic location. Three regions with the highest incidence rate and incidence rate in the capital city (Tbilisi) are presented on the line chart. The graph describes the increase in incidence in 2012–2013 years.

The regions of Kvemo-Kartli (n = 295, 43%), Kakheti (n = 106, 18%), Tbilisi (n = 65, 16%) and Samtskhe-Javakheti (n = 22, 9%), located in Eastern part of Georgia, accounted for 80% of the total cases. The annual incidence rate in Kvemo- Kartli was 4.72/100 000 in 2008. Then, the rate decreased to 2.0/100 000 in 2010, but increased to a peak of 18.58/100 000 in 2012 and remained at 12.33/100 000 in 2013. The rates in Kakheti follow a similar pattern to that in Kvemo-Kartli, except in 2015. The rates in Tbilisi and Samtskhe-Javakheti are generally under 1/100 000, except in 2015. In 2015, the incidence rate in Samtskhe-Javakheti increased to 6.24/100 000 (Fig 1.)

**Fig 2:**
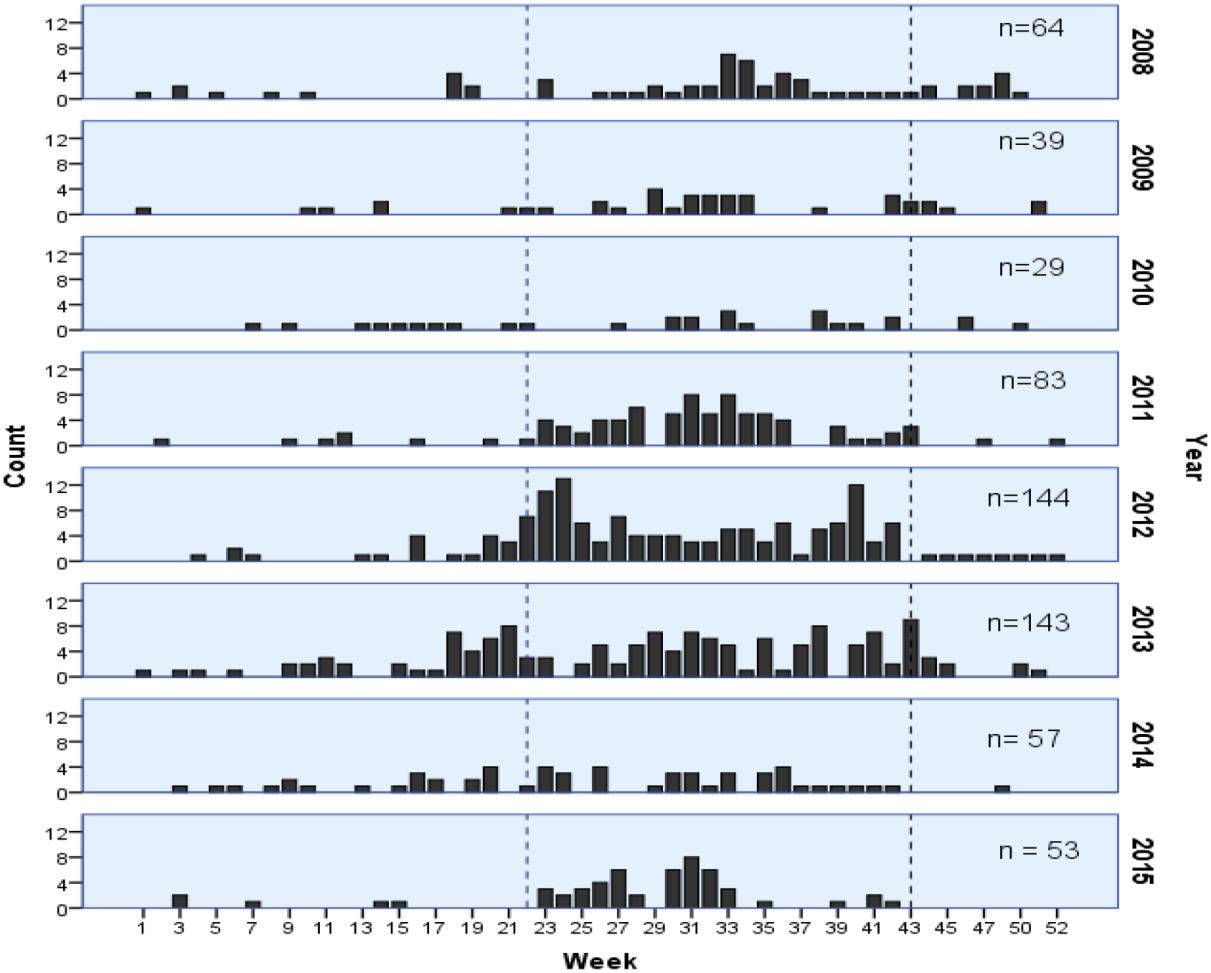
Annual cases of cutaneous anthrax by week in the year in Georgia (EIDSS 2008–2015). Variation in weekly counts of disease occurrence and shifts in seasonal cycles are presented on the figure.

In 2012–2014, compared to 2008–2011 and 2015 an increase in the notification started 13 weeks earlier, from late February instead of late May, respectively. Fig 2 demonstrates this shift in the notification of cutaneous anthrax cases. The ARIMA analysis confirms that statistical differences exist by year (b = 0.186, p<0.001) and by week in the year (b = 0.024, p<0.001).

Binary multiple logistic regression analysis of the risk factors for human cutaneous anthrax surveillance data from 2011–2015 showed the significant association of human cutaneous anthrax with handling of animal products (aOR 4.36, 95% CI 2.61–7.26), with living near pastoralist routes (aOR 2.74, 95%CI 1.57–4.76) (Table 3) and with travel to endemic areas within two weeks before the disease onset (aOR 2.32, 95%CI 1.14–4.69). Other variables included in the final model were: age group, ethnicity and earthwork.

**Table 3:** Binary multiple logistic regression analysis of risk factors for a confirmed/probable cutaneous anthrax.

(EIDSS 2011–2015, n = 768) Characteristics	Adjusted Odds Ratio (95%CI)
Age group	0.98 (0.73–1.31)
Living near pastoralist routes	2.74 (1.57–4.76)
Handling animal products	4.36 (2.61–7.26)
Ethnicity	
Georgian	1.33 (0.59–2.96)
Azerbaijani	0.68 (0.27–1.68)
Travel to endemic areas within two weeks before the disease onset	2.32 (1.14–4.69)

## Discussion

Our study describes the dynamics of human cutaneous anthrax in Georgia from 2008 to 2015 and associated major risk factors based on the analysis of the national surveillance data. National incidence rates of cutaneous anthrax in humans increased significantly in 2011–2013 and then declined in 2015. This finding is comparable to the finding in Turkey [12]. A comprehensive explanation for the observed rise and fall of the incidence rates of human cutaneous anthrax in the country of Georgia in 2008–2015 remains to be clarified but is likely associated with discontinuation of mandatory national livestock vaccination in 2008 coupled with weakened national human and animal health systems which were disrupted after the Soviet Union collapse [3]. Up to 2008, vaccination coverage was ~30–50% in cattle and small ruminants. Between the years 2009–2013, mandatory livestock anthrax vaccinations targeted ring vaccinations surrounding registered animal cases as well as voluntary vaccination by the farmer. The voluntary vaccination practice is still not culturally acceptable in the country of Georgia. The marked increase in animal and human cases stimulated the NFA to reinstitute, on a smaller scale, a national anthrax preventive vaccination campaign in the autumn of 2013. The program remains active to date and targets higher incidence districts twice a year in April and October but only covers ~15–20% of livestock. The combination of demographic and basic animal husbandry practices was strongly associated with cutaneous anthrax consistent with previous reports in Georgia [13], Turkey [12] and Kazakhstan [6]. Our study also suggests that human cases of cutaneous anthrax are usually secondary to an anthrax epizootic outbreak occurring in livestock and related to contact with infected animal products. Given this, food safety remains a concern. Spatial studies have previously reported clusters of cases of cutaneous anthrax located in towns along the animal migration corridors that spread from Kakheti near the border with Azerbaijan, through Kvemo-Kartli near Armenia and Turkey [14]. New research needs to estimate transmission potential of cutaneous anthrax cases along pastoralist routes, especially if those cases are pastoralists themselves. However, the current results highlight the importance of monitoring disease among population groups living nearby pastoralist routes and calls for the development of a novel surveillance program that will require additional screening methods to monitor pastoralists populations subject to higher risks of anthrax within and cross-border in Georgia.

Lastly, our study confirms that national incidence rates differ by year and by weeks in the year and apparent shifts in seasonal cycles in later years. Seasonal fluctuation is known in agricultural areas in Georgia that could partially explain the pattern of endemic cases of anthrax seen (Fig 2). Kracalik et al. have already reported associations with alkaline soil pH and ambient temperatures above 15°C [15], but the epi-curve (Fig 2) warrants further clarification. It is hypothesized that the first rise in cases occurs in the spring when animals are exposed to soil that has been disturbed during periods of increased rainfall, especially post-flooding. The soil is disturbed again in the fall during droughts when animals graze more closely to the ground thereby increasing their exposure to spores in the soil. The increase in soil movement increases the risk of infection in humans. Also, anthrax spores have been shown to be hydrophobic and are believed to rise to surface waters and collect on plants growing at the edges of ponds that progressively dry during the summer [16].

It is important to clarify whether the shift in an increase of cutaneous anthrax notification towards earlier in the year, in 2012–2014, can be explained by a combination of better reporting and a true difference in wet and dry periods. Therefore, additional research is recommended to build upon the work of Kracalik et al. 2014 [16].

Our study has limitations. We used routine surveillance data, and cases of human cutaneous anthrax might be underreported. Our analysis address occupational and pastoralist risk factors for cutaneous anthrax only in 2011–2015. Data on the risk factors for the years 2008–2010 are sparse in the EIDSS. Furthermore, we used rejected cutaneous anthrax cases from the surveillance database as a comparison group in our binary multiple logistic regression model. Future studies are proposed to examine temporal associations between the dates and locations of animal vaccinations and a corresponding reduction in human cases which permits better estimates of the true impact of animal vaccination program on human incident cases.

This study provides eight-year trends for cutaneous anthrax in humans in the country of Georgia. Our results underscore the importance of collaboration and evidence-based interventions undertaken by the Ministry of Labour, Health and Social Affairs and Ministry of Agriculture.

## Supporting information

S1 File Arima time series models. (SAV)

S2 File Logistic regression. (SAV)
