# Correction: Rates and risk factors for human cutaneous anthrax in the country of Georgia: National surveillance data, 2008–2015

**DOI:** 10.1371/journal.pone.0196958

**Published:** 2018-05-02

**Authors:** Ana Kasradze, Diana Echeverria, Khatuna Zakhashvili, Christian Bautista, Nicholas Heyer, Paata Imnadze, Veriko Mirtskhulava

The seventh author’s name is spelled incorrected. The correct name is: Veriko Mirtskhulava.
